# Maximizing the potential benefits of beaver restoration for fire resilience and water storage

**DOI:** 10.1002/eap.70102

**Published:** 2025-10-13

**Authors:** Jessie A. Moravek, Justin Brashares, Manuela Girotto, Randi Spivak, Andy Kerr, Andrea Molod, Shane Feirer, Robert Johnson, Augusto Getirana, Emily Fairfax, Albert Ruhí

**Affiliations:** ^1^ Department of Environmental Science, Policy, and Management University of California Berkeley Berkeley, California USA; ^2^ The Center for Biological Diversity Washington DC USA; ^3^ The Larch Company Ashland Oregon USA; ^4^ Global Modeling and Assimilation Office, NASA Goddard Space Flight Center Greenbelt Maryland USA; ^5^ University of California Agricultural and Natural Resources Berkeley California USA; ^6^ Hydrological Sciences Laboratory, NASA Goddard Space Flight Center Greenbelt Maryland USA; ^7^ Science Applications International Corporation Greenbelt Maryland USA; ^8^ Department of Geography, Environment, and Society and St. Anthony Falls Laboratory University of Minnesota Twin Cities Minneapolis Minnesota USA; ^9^ Present address: Department of Geography, Environment, and Society and St. Anthony Falls Laboratory University of Minnesota Twin Cities Minneapolis Minnesota USA

**Keywords:** beaver, BRAT model, *Castor canadensis*, drought, fire, Sierra Nevada

## Abstract

Restoring populations of native keystone species can increase landscape resilience to global change when those species create or modify ecosystems. The North American beaver (*Castor canadensis*) is an ecosystem engineer that increases river water storage and residence time, increasing fire resilience at the landscape level. Beaver populations in North America are significantly lower than they were historically, but over the last decade, beavers have been increasingly recognized for their ecosystem services, and reintroduction efforts throughout their historic range have become more prevalent. Here, we modeled potential beaver dam‐building capacity, associated surface water storage, and fire resilience in California's Sierra Nevada, a region at high risk of drought and wildfire. We estimate that 51% of beaver dam‐building capacity remains in this region compared to historical levels, and considerable dam capacity remains in all watersheds. Our conservative estimates suggest that beaver dams have the potential to store a total of 120 million m^3^ of surface water and create 2200 km^2^ of fire resilience in high fire risk areas. Additionally, streams where beavers have the potential to create the greatest water and fire benefits due to physical landscape and habitat characteristics are frequently found within watersheds that are at high risk for both drought and fire. Specifically, we identified five priority watersheds that have both high risk for drought and fire impacts, and have high potential to benefit from beaver conservation and restoration. Even in areas where fire and drought are less probable, the reestablishment of beavers will likely provide similar benefits. This unique approach to quantifying potential beaver benefits illustrates that wildlife can increase resilience to global change stressors and suggests that biodiversity and nature‐based climate solutions are intertwined.

## INTRODUCTION

A growing effort within biodiversity conservation is advancing the restoration of keystone and ecosystem engineer species, which can significantly modify the physical environment for their own benefit (Byers et al., 2006; Hale & Koprowski, 2018; Power et al., 1996). Restoring populations of these species has cascading effects on other animal and plant communities, alters landscape structure, and creates resilience to global change through a variety of biophysical processes (Byers et al., 2006; Yi & Jackson, 2021). Restoration of keystone species typically occurs in environments where they have the best chance of persisting, and/or in places where conflict with humans will be minimized. However, our awareness that restoring keystone wildlife species could help address major challenges associated with global climate change is increasing, specifically in the case of drought and wildfire. The potential for ecosystem engineers to function both as ecosystem modifiers and nature‐based climate solutions is a growing area of research and the motivation for this study.

The North American beaver (*Castor canadensis*) is a highly adaptable ecosystem engineer and the ideal case for considering how wildlife restoration can create resilience to climate change. Historically, beaver populations in North America ranged from 60 to 400 million prior to the fur trade, but current populations are thought to be 10–40 million (Brazier et al., 2021; Larsen et al., 2021; Naiman et al., 1988). Beavers use stones, mud, herbaceous material, riparian trees, and shrubs to build dams in stream channels that create beneficial pond and wetland habitat for themselves (Gurnell, 1998; Naiman et al., 1988). Beavers also dig channels that fan out into the surrounding floodplain area and across valley bottoms, which help them more easily navigate their territories from the safety of the water and contribute to water spreading throughout the floodplain (Fairfax & Whittle, 2020; Grudzinski et al., 2020; Hood & Larson, 2015). Dam‐building and channel‐digging activity by beavers are defining processes in aquatic and riparian ecosystems. Beaver ponds change riverine hydraulic geometry by widening channels and increasing their wetted surface, slowing down water, and changing sediment transport processes in a way that helps reduce stream incision and the frequency of bankfull floods (Pollock et al., 2007, 2014). Beaver dams can also alter nutrient cycling by increasing residence time and water temperature, and by storing both nutrients and sediments (Wohl, 2021). Beaver dam complexes also have landscape‐level effects by increasing riparian vegetation wetness and landscape resilience to wildfires (Fairfax & Whittle, 2020), and mitigate floods and droughts (Hood & Bayley, 2008; Ronnquist & Westbrook, 2021; Westbrook et al., 2020).

Critically, beaver dams slow the movement of water through the landscape (Brazier et al., 2021; Gurnell, 1998; Westbrook et al., 2006) by storing water in surface and subsurface habitats and by creating landscape “roughness,” which slows water as it moves around, through, over, or under beaver dams (Green & Westbrook, 2009; Jordan & Fairfax, 2022; Puttock et al., 2017). Medium‐sized beaver pond complexes have been shown to store up to 1000 m^3^ of water (Puttock et al., 2017), which leaks out of the dam slowly over time, buffering variation in streamflow even during dry seasons (Majerova et al., 2015; Puttock et al., 2017). Reintroduced beavers can turn intermittent streams into perennial streams that flow year‐round, which can have ecological impacts (e.g., for native desert fishes adapted to flow intermittency, Gibson & Olden, 2014). However, beaver‐mediated changes in the water cycle are generally considered to be beneficial in terms of reconnecting streams with groundwater and floodplain habitats (Pearce et al., 2021).

Beaver dams also create fire resilient landscapes. By building dams, digging channels, raising water tables, and reducing stream incision, beavers create zones of wet, well‐connected floodplains (Jordan & Fairfax, 2022; Weirich III, 2021; Whipple, 2019). A study of wildfires in the western United States determined that stream segments with beaver activity maintained significantly higher vegetation greenness during and after fire than stream segments without beavers, indicating increased fire resilience associated with beaver ponds (Fairfax & Whittle, 2020). Similarly, a study in the Rocky Mountain region found that beaver ponds decreased burn severity in surrounding floodplain areas during megafires (Fairfax et al., 2024). Even when beavers are not present, abandoned beaver dams can help with fire recovery. Studies in the Rocky Mountains quantified how, after fires, abandoned beaver dams can trap sediment and promote overbank flow, which reduces bank erosion and facilitates postfire floodplain vegetation regrowth (Wohl, 2021). Active beaver ponds, especially those that are larger and older, can also trap large amounts of postfire sediment and prevent it from damaging downstream ecosystems (Dunn et al., 2024). Based on these findings, beavers are increasingly recognized for their potential to create networks of fire‐resistant wetlands, potentially slowing the spread of fires and giving humans more time to mobilize firefighting resources (Fairfax & Whittle, 2020; Jordan & Fairfax, 2022).

Although beavers were historically widespread throughout North America, by 1900 populations had been severely depressed by the industrialized fur trade (Naiman et al., 1988). Over the last several decades, beaver populations have been recovering in much of North America, although beaver populations remain at about 10% of historical levels in the United States (CDFW, 2023; Naiman et al., 1988). Similar to the rest of North America, beavers were once common in most of California, but populations have been slow to recover and are currently low throughout the state (CDFW, 2023). As California landscapes and communities are increasingly susceptible to drought (Berg & Hall, 2017; Diffenbaugh et al., 2015; Liu et al., 2022) and wildfire (Brown et al., 2023), evidence of beavers' role in mitigating these stressors has led to renewed investment in beaver restoration by the state of California (Castaneda, 2023; CDFW, 2023). Water storage and fire resilience solutions are high priorities for land and water managers across the state, and acceptance of beaver restoration, both politically and socially, is growing. Developing a robust scientific understanding of the potential for restoring beaver dam‐building activity, and how that dam‐building activity could confer water storage and fire resilience benefits on a landscape scale, is necessary for informed beaver management decision‐making.

The goal of this study is to quantify the potential opportunities and benefits of restoring beaver dam‐building activity to reduce some of the consequences of global climate change. We focused our study on the Sierra Nevada region of California, where water shortages and extreme wildfires are critical issues (Ahmad et al., 2024) and where beaver reintroduction efforts are already underway (Castaneda, 2023). Our results can immediately inform future beaver restoration priorities in this region. We examined a large, multi‐watershed region and evaluated multiple beaver‐related impacts, culminating in a unique quantitative perspective on the potential climate and water‐related benefits of beaver restoration in this area. We modeled current beaver dam capacity in the Sierra Nevada and determined how much dam‐building capacity has changed in comparison to historical levels. We also measured the extent to which restoring dam‐building activity could provide potential water storage and fire resilience benefits across the region and evaluated how these potential benefits co‐occur on the landscape. Not all beavers engage in dam‐building activity, instead opting to create bank burrows, especially in large rivers or lakes where building a dam would be challenging (Brazier et al., 2021). However, in this study, we focused on dam‐building activity because it has the most direct and readily measurable effect on water storage and is often co‐located with beaver‐dug channels, tree chewing, and pond construction.

Here, we hypothesized that current beaver dam capacity would be significantly lower than historical dam capacity due to extensive beaver trapping and large‐scale land use changes over the last century that resulted in riparian habitat degradation and increased human‐wildlife conflict, but that considerable dam‐building capacity would remain throughout the region. Additionally, we expected that (1) streams with high potential for beaver dam‐building activity would sometimes coincide with high fire risk areas, creating the potential for enhanced fire resilience, and (2) reaches with high potential for beaver‐related surface water storage would sometimes coincide with high water‐scarcity watersheds, creating the potential for enhanced drought resilience. We commented on other considerations (land ownership, biodiversity of wetland species, livestock grazing practices) for prioritizing beaver reintroduction and conservation. Finally, we identified areas where potential fire and water benefits from beaver overlap in high‐risk watersheds, and thus where beaver restoration has the greatest overall potential as a nature‐based climate solution.

## METHODS

### Study site

**FIGURE 1:**
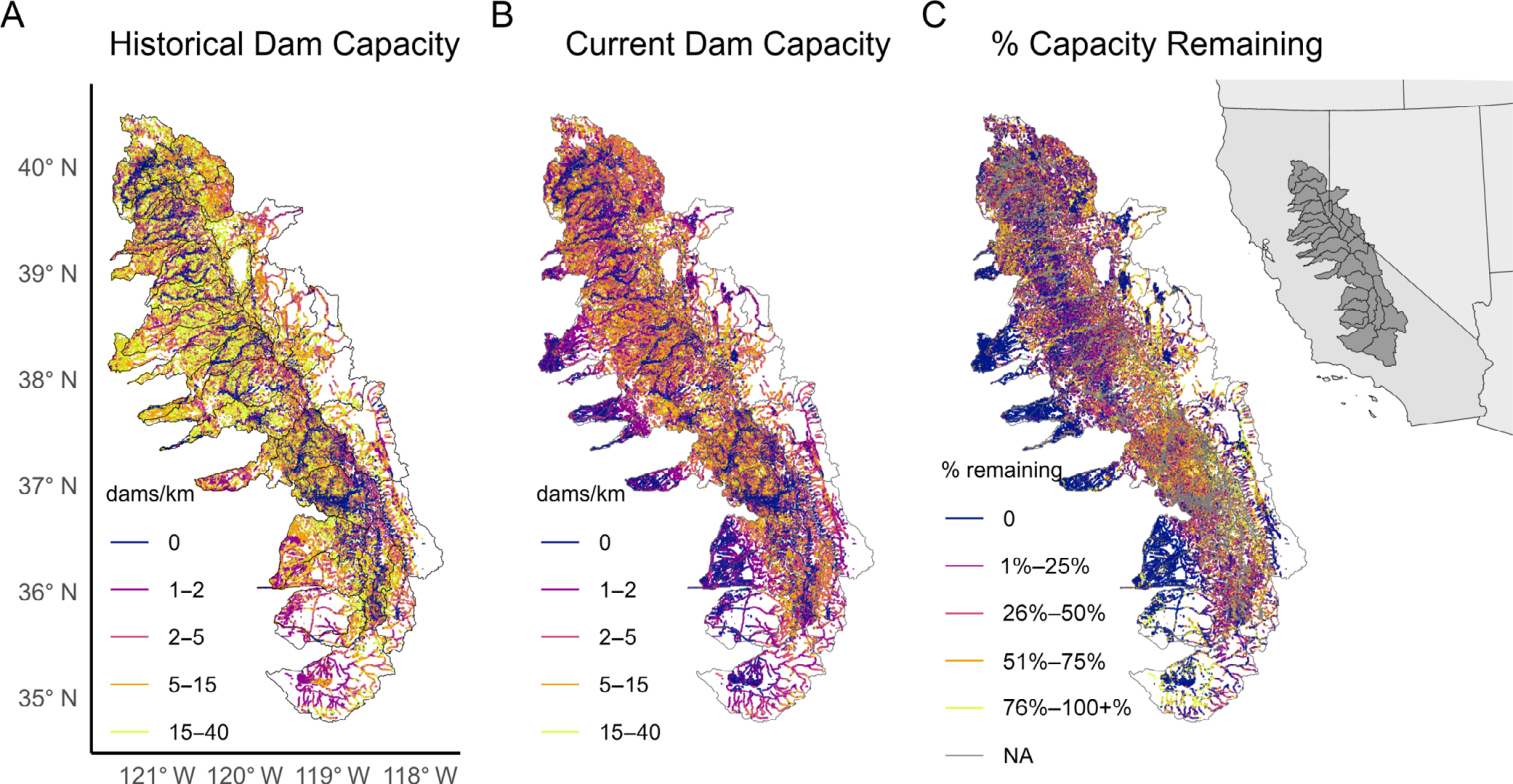
Change in beaver‐built dam capacity in 159,000 km of streams in the Sierra Nevada region (see inset) over time. (A) Historical beaver‐built dam capacity (in no. dams per kilometer) in the Sierra Nevada region, based on historical vegetation data. This region could have supported about 897,000 dams historically. (B) Current beaver‐built dam capacity (in no. dams per kilometer) in the Sierra Nevada region. The region can currently support about 440,000 dams. (C) Change in dam capacity between historical and current times. Dam capacity in the region has declined about 50% compared to pre‐European settlement, but significant dam capacity remains in every watershed in the region. NA, not applicable.

The Sierra Nevada mountain range spans much of eastern California and parts of western Nevada (Figure 1). The Sierra Nevada Mountains have cool, wet winters and warm, dry summers, and almost all precipitation occurs between October and May (Melack & Stoddard, 1991). In higher elevations, most precipitation falls as snow that starts melting in spring. The windward western Sierra receives about ⅔ more rain than the leeward eastern slopes. Vegetation is diverse and highly dependent on elevation, but generally consists of a combination of alpine meadow and pine forest at higher elevations, turning to deciduous oak forest in foothill regions, with riparian areas populated by willows and aspens (Landfire, 2021).

Current beaver population size and distribution in the Sierra Nevada mountains is unknown. For most of the 20th century, beavers were widely considered non‐native above 300 m in the Sierra Nevada region (Lanman et al., 2013). However, Indigenous peoples in the Sierra Nevada region have long understood beavers to be a critical part of the local ecosystem (Keeble‐Toll, 2018; Sherriff, 2021). Based on traditional ecological knowledge from Indigenous groups across California, as well as archeological evidence from ancient beaver dams, beavers are now recognized as a native species to the Sierra Nevada region (James & Lanman, 2012). In 2023, the Mountain Maidu Summit Consortium and Tule River Tribes spearheaded the first beaver reintroductions in the Sierra Nevada for over 75 years (Castaneda, 2023). Other than the individuals released in this reintroduction, a few beaver colonies exist, notably in the Lake Tahoe region and the southern end of the range (Fairfax et al., 2023), but generally, beaver populations in the Sierra Nevada today are significantly smaller than those before European colonization (James & Lanman, 2012).

Here, we defined the Sierra Nevada using regional classifications from Zimmerman et al. (2018), and focused on 31 USGS hydrologic units (HUC‐8) watersheds overlapping with the region (Figure 1). Although parts of many of these watersheds extend outside our definition of the Sierra Nevada region, we included full HUC‐8 catchments to understand how beaver dam capacity dynamics might vary throughout a watershed spanning different regions. We excluded watersheds that were outside the confirmed historical range of beavers in California (e.g., watersheds in southern California such as the Antelope‐Fremont Valleys watershed), even if those watersheds fall within the Sierra Nevada region and currently have beaver within them (Lanman et al., 2013).

### Modeling framework overview

**FIGURE 2:**
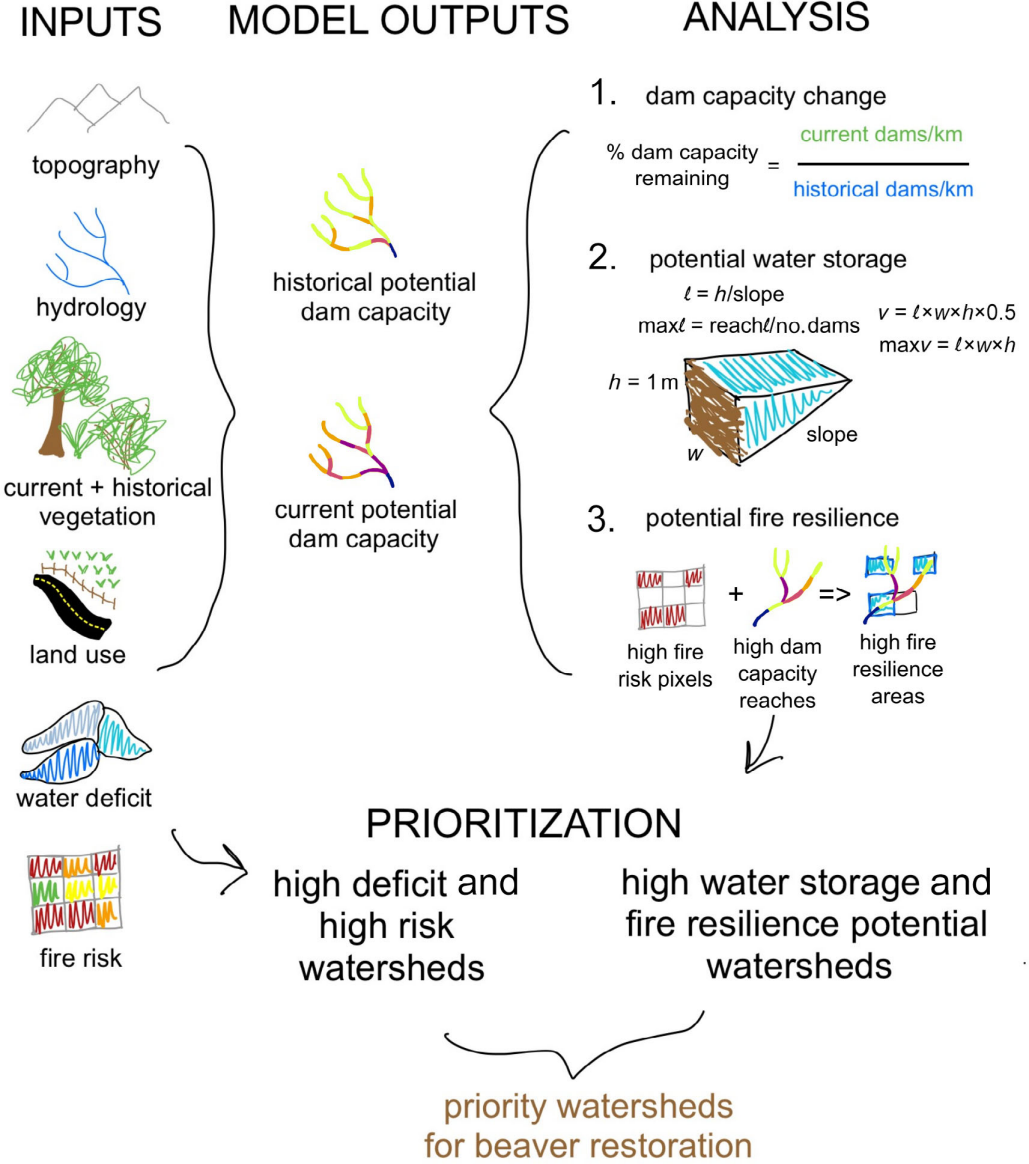
Schematic methods diagram of data inputs, model outputs, and analyses, described in *Methods*. Figure by Jessie A. Moravek.

This study combines results from several models, as schematized in Figure 2. We first modeled historical and current potential beaver dam‐building capacity (see BRAT model in the next section). We used those model results to estimate current potential surface water storage and quantified potential fire resilience at the reach scale by identifying areas with both high fire risk and high potential dam capacity. We then identified “priority” watersheds, or watersheds with high water‐scarcity and high fire risk, where beaver‐related water and fire benefits could have the most impacts. Finally, we overlaid potential water storage and fire resilience to identify areas where beaver restoration would create both benefits at the same time.

### Beaver dam‐building capacity

The Beaver Restoration Assessment Tool (BRAT) model was developed by Macfarlane et al. (2017) to estimate the maximum capacity of a stream network to support beaver dam‐building activity. This model uses a fuzzy inference system built upon known habitat requirements of beavers to integrate topography, hydrology, vegetation, and land use spatial datasets, identifying physical and ecological conditions that would allow beavers to successfully build and maintain varying numbers of dams (Figure 2). The beaver dam‐building capacity output provides valuable information for understanding where and how to restore beaver populations. We obtained all data from publicly available national datasets (as described next) and used pyBRAT 3.1 with modifications for use in ArcGIS Pro 3.1.

We used a ⅓ arcsecond (10 m) digital elevation model (DEM) from The National Map (US Geological Survey, 2024a) to calculate the valley bottom footprint using the Valley Bottom Extraction Tool (VBET, Gilbert et al., 2016), which we hand‐edited for accuracy, as well as stream channel slope and drainage area.

We mapped stream networks using the National Hydrography Dataset (NHD, US Geological Survey, 2024b) and excluded those marked as ephemeral, because these streams likely do not have enough streamflow to regularly support beaver dams. We also excluded streamflow patterns marked as “unknown” because no information was available in the dataset about what portion of the year these streams contained water (US Geological Survey, 2023). The resulting network included only perennial and intermittent streams and canals. We divided the stream network into 300‐m segments and represented discharge using regional regression equations for a 2‐year flood and baseflow. We used 2‐year flood equations for hydrological regions that encompassed each watershed (Gotvald et al., 2012). Baseflow equations were calculated based on watershed‐specific variables for each watershed (Riverscapes Consortium, 2018). We calculated elevation, precipitation, and slope for each watershed from USGS StreamStats (US Geological Survey, 2019).

We used both historical and existing vegetation rasters from the Landfire dataset in our models (Landfire, 2021). For existing vegetation, we used the Landfire Existing Vegetation Type layer from 2020, and for historical vegetation, we used the Landfire Biophysical Settings included in the 2016 dataset (the biophysical settings dataset represents modeled estimates of vegetation type prior to European settlement in North America. The most recent biophysical settings model available is from 2016; Blankenship et al., 2015, 2021). We edited the attribute table of each raster to include a vegetation suitability index for beaver dam building (Macfarlane et al., 2017). We coded each vegetation type varying from 0 to 4, with 0 being no suitability and 4 being the best suitability. For example, vegetation like aspens and willows are known preferred food and building materials, and thus are considered highly suitable habitats for beavers and were coded as 4, while exposed rock, glacier, or cropland where beavers could not persist were coded as 0. Beavers do occasionally eat crops, especially trees associated with orchards, but these materials are generally poor for dam building, and crop habitat is unsuitable for beavers from a human‐wildlife conflict perspective. The BRAT model bases historical beaver dam capacity estimates on the Landfire biophysical settings dataset, which reflects modeled vegetation pre‐European settlement. The model does not alter hydrology or topography, the other main physical drivers of dam capacity, to make historical estimates, meaning that all modeled dam capacity changes between historical and current levels are the result of vegetation change. Hydrology in California has changed significantly since pre‐European settlement in ways that are likely meaningful for beaver, but those hydrological changes are complex and beyond the scope of this study other than the identification of canals and human‐made reservoirs.

To represent land use, we edited the attribute table of the existing vegetation raster to include a land use code, following Macfarlane et al. (2017). The land use code ranges from 0 to 1 and represents very low to very high human land use. For example, the very low human land use category includes natural settings with limited land use, and the very high land use category includes urban areas. We also included road and railroad layers from the US Census Bureau TIGER/Line dataset (United States Census Bureau, 2023). We created a human‐made canal layer by selecting streams coded as canals in the NHD (US Geological Survey, 2023). We applied a land ownership layer from the Bureau of Land Management Surface Management Agency dataset (2024). We used a protected area layer and a conservation easement layer from the protected area database of the United States (US Geological Survey, 2024c). These anthropogenic features and influences can either limit or enhance dam capacity by altering habitat suitability for beavers.

### Estimating potential surface water storage in beaver ponds

Using dam‐building capacity outputs from the BRAT model, we estimated surface water stored above each potential beaver dam. Beaver wetlands also store water in soil and groundwater, but these are beyond the scope of this study, meaning that the surface water storage we quantify here is a conservative estimate of water storage. Following a method developed by Scamardo et al. (2022), we approximated beaver ponds as right triangular prisms. We assumed that beaver dams were 1 m tall, although actual dam heights can vary widely (Hafen et al., 2020; Figure 2). For simplicity, we assumed that pond width was equal to channel width (i.e., no major flooding outside the stream channel; this is a conservative estimate because flooding or ponding often occurs in widened inundated areas behind beaver dams), and estimated channel width based on national hydraulic geometry relationships (Wilkerson et al., 2014). We calculated pond length as a function of slope and dam height, and we set a maximum pond length for reaches with multiple beaver ponds by dividing the length of the reach by the number of potential dams in that reach. If calculated pond length exceeded the maximum pond length for that reach, we used maximum length in calculations. We calculated maximum pond volumes by taking the volume of a triangular prism (Equation 1):

(1) Pond volume=damheight×channel width×damlength×0.5

We calculated water deficit, a proxy for drought risk, for each watershed using the Normalized Deficit Cumulated (NDC; Devineni et al., 2015). The NDC is the maximum cumulative deficit between average water demand and renewable water supply, divided by rainfall volume, for each county. NDC only includes internal sources of renewable water and excludes rivers and canals flowing through the county, therefore reflecting how much each county relies on nonrenewable or external water sources. We calculated HUC‐8 level NDC by taking the percentage of each county in each watershed and multiplying by the NDC of that county, treating NDC as cumulative over space (as in Ruhi et al., 2018; US Geological Survey, 2019).

### Estimating potential fire resilience conferred by presence of beaver ponds

To quantify the spatial distribution of fire risk, we used a continuous raster of wildfire hazard potential (WHP) at a 270‐m resolution (US Forest Service, 2020, Appendix S1: Figure S1). WHP values are based on the presence of fuels with the potential for extreme fire behaviors such as torching and crowning, and this dataset is generally used for targeting long‐term vegetation management. Although in this study we refer to WHP as “fire risk” for clarity, WHP values do not take into account weather forecasts or moisture conditions and therefore are a static map that does not reflect actual fire risk for any specific day or season (US Forest Service, 2020); it is instead a broad strokes indicator of ongoing risk. The WHP values ranged from 0 to 40,000, and we classified pixels as “high risk” if WHP > 2000, which is the threshold for the top quartile of WHP values in this region. Like the BRAT model, the WHP values were calculated based on the 2020 Landfire vegetation dataset (Landfire, 2021; US Forest Service, 2020).

To quantify the potential fire resilience conferred by beaver dams, we focused on areas with both high WHP and high modeled dam capacity. We identified high‐risk–high‐capacity areas by overlapping raster cells with WHP > 2000 and stream segments where potential beaver dams per kilometer >5. We quantified the percentage of stream network that was high‐risk–high‐capacity in each watershed by dividing total high‐risk–high‐capacity area by high‐risk fire area along streams (we restricted this calculation to streams because potential beaver activity is also restricted to streams). We expect that our estimates of high‐risk–high‐capacity areas provide a conservative estimate of the actual fire resilience benefits that would be conferred by beavers. Previous research on megafires in the Rocky Mountains has shown that any number of beavers present on a landscape confers fire resilience, regardless of the BRAT‐modeled dam capacity of the stream, and that fire‐resistant area often extends far beyond the ponds, likely due to the influence of beaver‐dug channels increasing wetted floodplain area (Fairfax et al., 2024).

### Watershed prioritization

**FIGURE 3:**
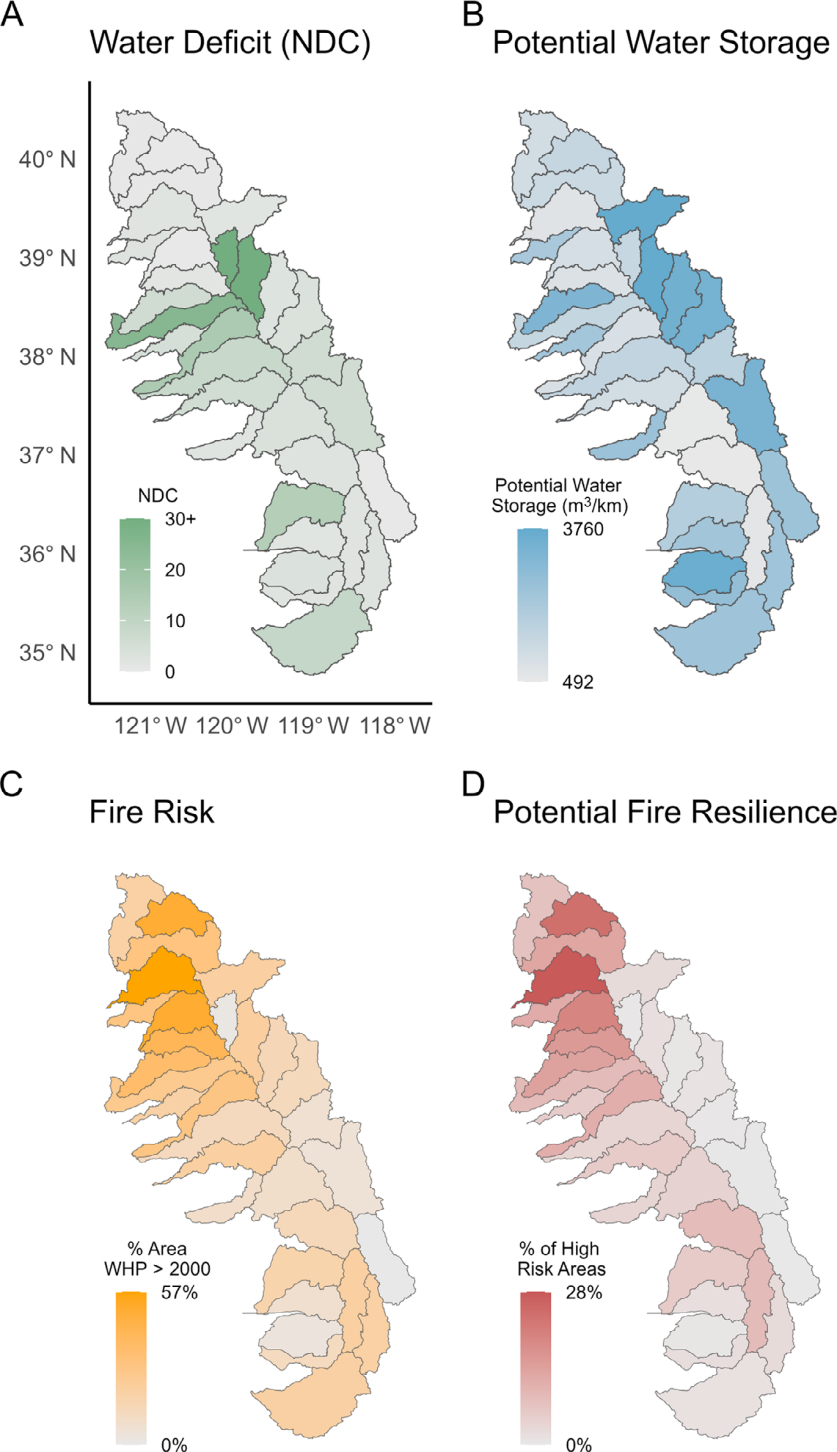
(A) Watershed‐scale water deficit (Normalized Deficit Cumulated [NDC]); (B) normalized potential water storage as calculated by cubic meter per stream kilometer for each watershed; (C) fire risk, as calculated by percent area of watershed with a wildfire hazard potential (WHP) >2000; (D) potential fire resilience, as calculated by percent area with high‐risk–high‐capacity overlap, restricted to stream corridors.

To quantify potential beaver‐related benefits in each watershed, we summarized historical dam capacity, current dam capacity, percent historical capacity remaining, water deficit, fire risk, potential water storage, and potential fire resilience area for each of the 31 watersheds in our study region (Figure 3, Appendix S1: Table S2). We defined high‐risk watersheds by identifying watersheds with both a water deficit (NDC > 1) and more than 20% of land area at high fire risk (WHP > 2000). We also overlaid fire risk with potential water storage to identify locations in each watershed where beaver restoration has the potential to support fire resilience, water storage, or both. For this overlay, we used only fire risk pixels that overlapped stream segments included in our study. We categorized potential water storage and potential fire resilience equally based on statistical quartiles. We identified watersheds with high proportions of overlapping co‐benefits that were also high‐risk watersheds. We also quantified the amounts of potential beaver dams, water storage, and fire resilience on private lands versus public lands (Appendix S1: Table S4). All analyses were performed in R (R Development Core Team, 2024).

### Uncertainty and bias

We acknowledge that BRAT‐derived estimates may be affected by uncertainty in the input data, and that limitations inherent to any geospatial modeling (e.g., due to data scale) likely led to an underestimation of associated benefits of beaver dams. With regard to uncertainty, BRAT uses fuzzy inference and combines lines of evidence (i.e., multiple variables that are known to promote or discourage beaver establishment) with rule tables. Uncertainty is accounted for by considering all inputs as continuous variables, rather than mutually exclusive (yes‐no) overlapping conditions. Based on aggregated membership functions, BRAT then predicts dam capacity for each stream segment, using centroids to assign a single value per segment (Macfarlane et al., 2017). Our estimates of potential surface water storage and potential fire resistance rely on this centroid value.

With regard to bias, we made decisions throughout the modeling exercise that would consistently lead to conservative estimates of estimated dam capacity and potential benefits provided by the beaver dams. We note that BRAT has not been fully validated for the state of California, but BRAT validation efforts in Montana show that BRAT underestimates beaver dam capacity about 18% of the time, usually because of dam building on floodplains or side channels that are not included in the stream network (Macfarlane et al., 2024). Preliminary validation work in four of our watersheds in the Sierra Nevada region (HUC 18030003, 18030002, 18020123, and 18090101) suggests that BRAT tends to underestimate beaver dam capacity (average: 50%) in stream segments where beavers persist (Appendix S2: Table S1, Figure S1; J. Moravek, unpublished data). The stronger underestimation of beaver dam capacity in California relative to Montana likely indicates that beavers in California may be using nontypical building materials and persisting in suboptimal habitats.

Additionally, because of the highly adaptable and unpredictable behavior of beavers beyond the reach scale represented in the BRAT model, our water and fire estimates are designed to be conservative to account for variability in beaver dam‐building behavior. To estimate potential surface water storage, we modeled beaver ponds as right triangular prisms, which do not account for water ponding outside the width of the channel. The exact area and volume of ponded water at a particular beaver dam are hard to define because it is highly dependent on valley bottom morphology, and because beaver dam‐building behavior is dynamic and unpredictable past the linear predictions at the reach scale used in the BRAT model. Finally, when estimating potential fire resistance, we only considered pixels that overlapped directly with stream corridors instead of the entire valley bottom. Beaver occupancy of valley bottoms is complex and not well understood, and because of this, BRAT estimates linear dam density along stream corridors (not laterally, across valley bottoms). Studies have shown that where beavers do occupy an entire valley bottom, they create almost complete fire resistance (Fairfax et al., 2024). Thus, our channel‐constrained fire‐resistant pixels are most likely an underestimate. For all these reasons, we think that our exercise provides a lower bound for potential beaver benefits across the Sierra Nevada. Subsequent exercises that improve scale and hydro‐ecological realism will most likely alter our estimates of beaver‐associated fire and water resilience.

## RESULTS

### Change in dam capacity from historical levels

Our calculation of current and historical beaver dam‐building capacity for 159,000 km of stream in the Sierra Nevada region determined that historically, the region could have supported 897,000 dams (Figure 1A; Appendix S1: Table S1), and it currently can support 51% of the historic estimate, or 440,000 dams (Figure 1B; Appendix S1: Table S1). Every watershed has experienced notable declines in dam capacity compared to pre‐European settlement (Figure 1C; Appendix S1: Table S2), but considerable dam capacity remains across the region. The BRAT model relies entirely on vegetation change to estimate dam capacity in historical versus current time periods. Major vegetation shifts related to agricultural or urban development have occurred in this region since historical times, leading to a decline in beaver‐favorable vegetation in most watersheds (Appendix S1: Figure S2; Landfire, 2021).

### Potential benefits of beaver dam restoration given current dam capacity

We found that in total, potential beaver dams in the study area could store up to 120 million m^3^ of water across the whole region (Figure 3B). For context, Mono Lake, which is a critical water source for the city of Los Angeles, has a water volume of approximately 3 billion m^3^ after diversions (Stine, 1991). In terms of water deficit, watersheds with the highest water deficits sometimes, but not always, overlapped with streams with the highest water storage (Figure 3A,B).

We found that potential beaver dams conferred a total of 2200 km^2^ of fire resilience in areas with high fire risk (Figure 3C,D). For comparison, this is slightly more than the total area burned by the 2024 Park Fire (1739 km^2^), which was one of the largest fires in California's history (CalFire, 2024).

### Identifying priority watersheds for beaver restoration

**FIGURE 4:**
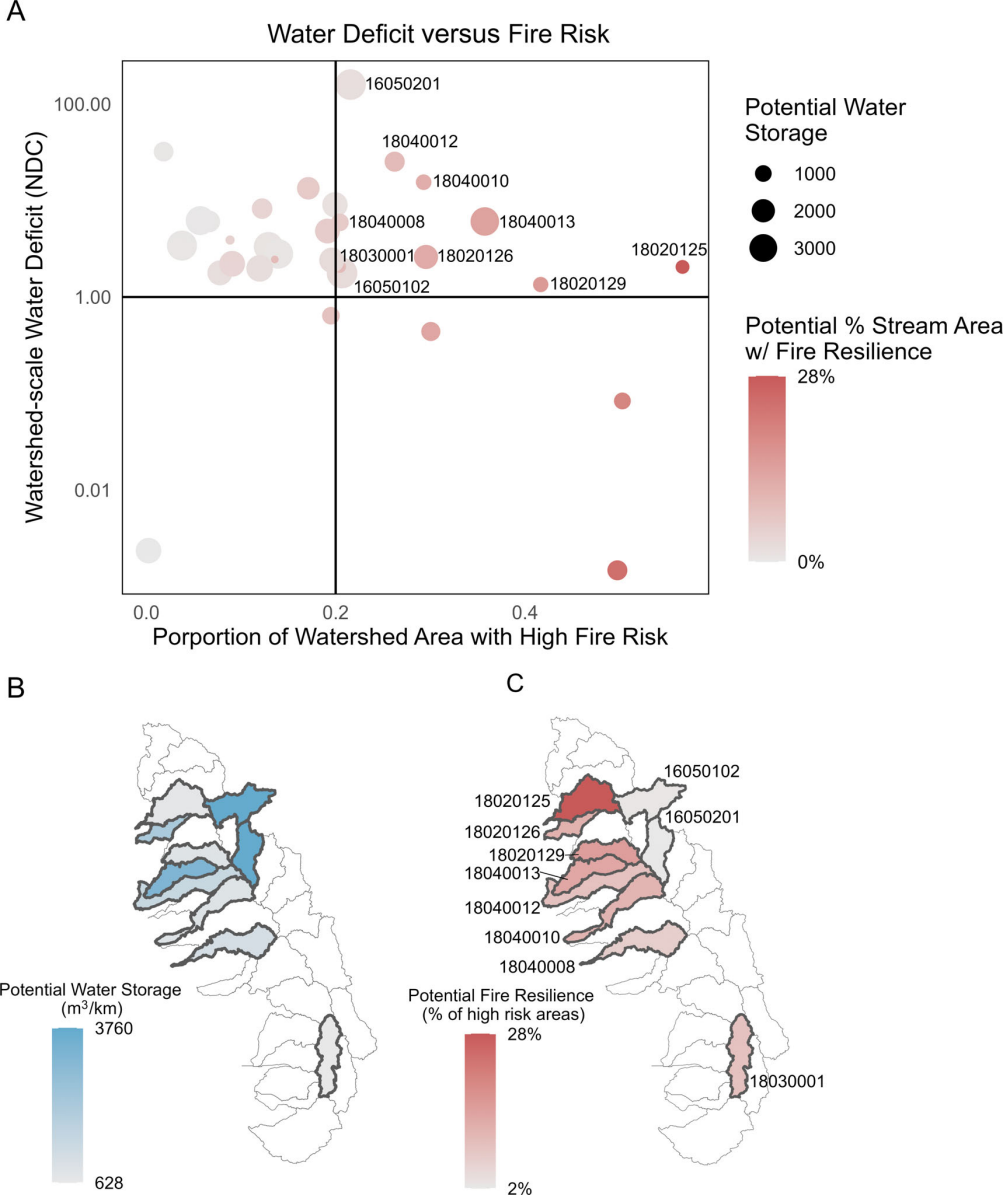
(A) We identified high‐risk watersheds by identifying watersheds with a water deficit (Normalized Deficit Cumulated [NDC] > 1), and watersheds with more than 20% of area at high fire risk (wildfire hazard potential [WHP] > 2000). We found 10 watersheds with both high NDC and high fire risk. These 10 high‐risk watersheds had varying (B) potential water storage and (C) potential fire resilience (for watershed HUC8 codes and names, please see Appendix S1: Table S2).

We identified 10 watersheds at the highest risk of experiencing both water deficit and fire at the watershed scale (Figure 4A). Of those 10 watersheds, potential water storage from beaver dams ranged from 628 to 3760 m^3^ (mean cubic meter per stream kilometer per watershed, Figure 4B). Potential fire resilience gained by projected beaver restoration ranged from 2% to 28% of each watershed (% high‐risk–high‐capacity area/high‐risk area, Figure 4C).

**FIGURE 5:**
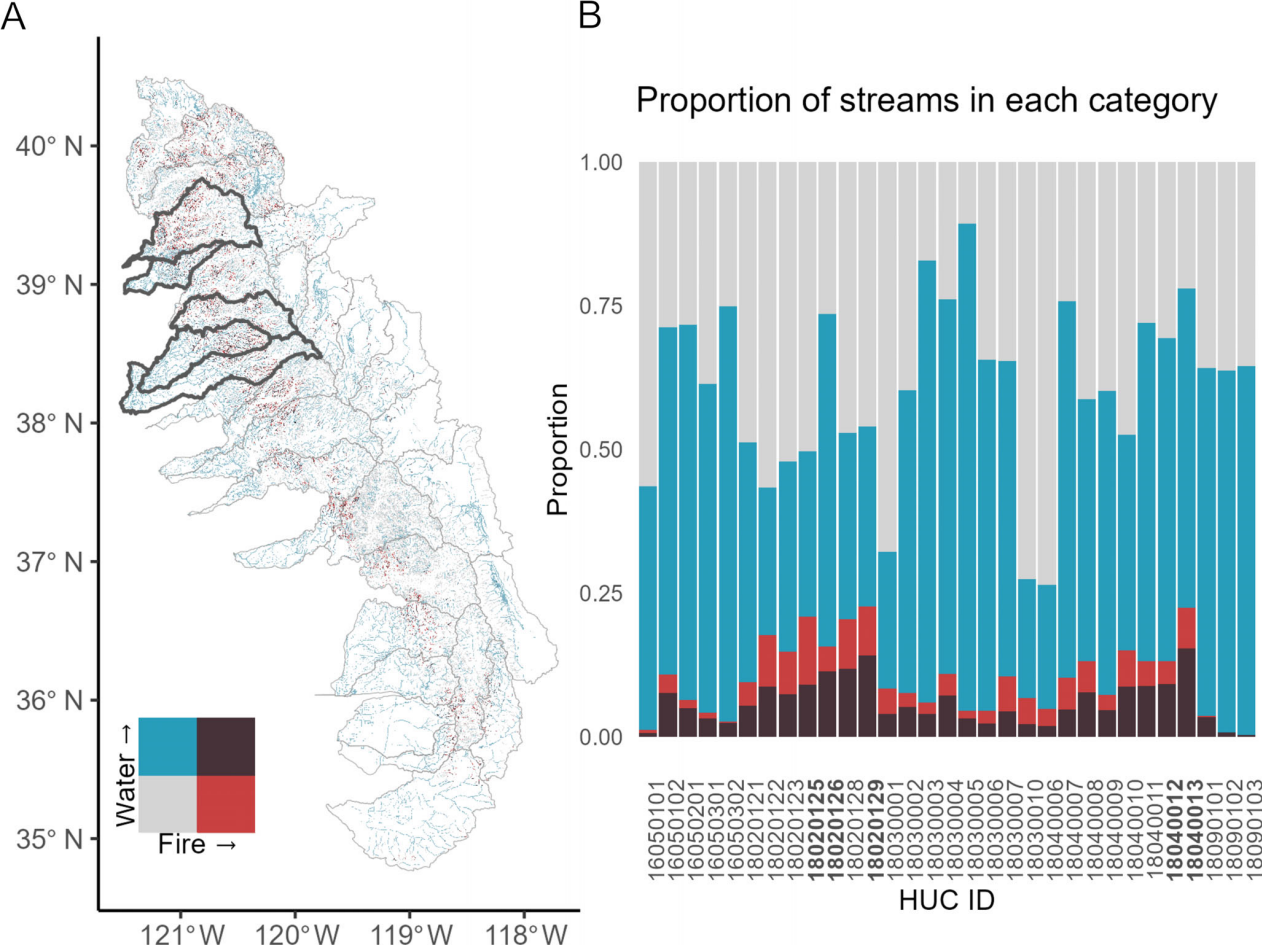
(A) Map of the region showing areas that prioritize potential water storage, fire risk, or both. We have categorized water storage and fire risk equally based on statistical mean. Highlighted watersheds represent five watersheds that we identified as high‐risk in Figure 4, and that have the most potential (i.e., upper quartile) for both fire and water benefits from beavers. (B) We use a bar plot to illustrate the proportion of stream network in each watershed that has potential for beavers to store water, create fire resilience, both, or neither. Bolded HUC8 codes represent 5 watersheds that we identified as high‐risk in Figure 4 (18020125—Upper Yuba; 18020126—Upper Bear; 18020129—South Fork American; 18040012—Upper Mokelumne; 18040013—Upper Cosumnes), and that have the most potential for both fire and water benefits from beavers (tallest dark red bars).

A second approach to prioritize beaver restoration is to look at a stream‐reach scale to identify where potential water storage and fire resilience from beaver dams overlap. We overlaid potential water storage with fire risk constrained to streams to identify areas of overlap (Figure 5A). For this analysis, we used high fire risk areas (WHP > 2000) instead of potential fire resilience created by beavers (WHP > 2000 and beaver dam capacity >5 dams/km) because beavers create fire resilience wherever they are present on the landscape (Fairfax et al., 2024). We found that of the 10 high‐risk watersheds (Figure 4), five had more than 8.7% (the third quartile) of stream area categorized as overlapping high fire risk and high potential for beaver‐related water storage. We identified these as high priorities for beaver restoration.

## DISCUSSION

We sought to estimate historical and current beaver dam‐building capacity in the Sierra Nevada region, quantify potential fire resilience and water storage benefits from beaver dams, and identify priority areas for beaver restoration based on potential to provide fire and water benefits. We determined that current beaver dam‐building capacity in the Sierra Nevada region has declined approximately 50% compared to historical (i.e., pre‐European) capacity, but the region retains considerable dam‐building capacity across all 31 watersheds (Figure 1). Beaver restoration has the potential to increase water storage and fire resilience across the region, but the levels of potential water storage and fire resilience conferred by beaver dams varied by watershed (Figure 3). We identified the most at‐risk watersheds with the highest water deficit and fire risk and described the potential of beaver dams to store water and create fire resilience in those areas (Figure 4). We also identified watersheds with the most potential for overlapping water storage and fire resilience (Figure 5). Overall, restoring beaver populations and beaver dam‐building capacity is a promising component of water and fire resilience plans in the Sierra Nevada region, providing an example of how reintroducing and conserving a native ecosystem engineer can help create resilience to climate change.

### Beaver dam capacity remains in all watersheds across the Sierra Nevada region

California's vegetation has undergone extreme change over the last three centuries, largely due to agricultural development (Shelton, 1987). Conversion from natural landscapes to agricultural landscapes has significantly decreased vegetation suitability for beavers. The BRAT model scores all agricultural land use and agricultural vegetation types (such as orchards, crops, vineyards) as “0” or unsuitable for beavers, partially because some of these landscapes cannot sustain beaver populations and partially because beaver presence on highly agricultural landscapes can cause conflict (Macfarlane et al., 2017). Many of the watersheds in this study on the western side of the Sierra Nevada range extend into California's Central Valley, which is a highly agricultural area, and conversion of vegetation in this and other parts of the study region has led to a decline in vegetation suitability for beavers and a decline in potential beaver dam capacity throughout the region (Appendix S1: Figure S2). However, although beaver‐friendly vegetation has declined across the region, riverscape restoration and native riparian plantings in some areas may improve habitat suitability for beavers and increase the potential area of drought and fire resistance over time. Conversely, beaver‐friendly vegetation types can be reduced by fire and human development.

Despite extensive land use change, considerable beaver dam‐building capacity remains in every watershed (Figure 1). Beavers are highly resilient and adaptable, and many areas retain the potential to sustain more than five beaver dams per stream kilometer, which would have considerable impacts on the ecosystem (Dittbrenner et al., 2022; Puttock et al., 2017). In the Sierra Nevada, areas with the highest dam capacity tend to be concentrated in headwater streams, which are at higher elevations and tend to be in protected areas such as National Parks or National Forests. On the other hand, larger rivers that are too wide and swift, or slope‐limited stream corridors that are too steep for beavers to maintain dams, have zero dam capacity (Macfarlane et al., 2017). The Central Valley region also has many areas with zero beaver dam capacity, largely because of agricultural landscapes that would be unsuitable for beavers (Macfarlane et al., 2017).

We note that our analysis includes perennial and intermittent streams, with 47.5% of the streams included in our analysis identified as intermittent (Appendix S1: Figure S3). We found that 23% of potential water storage and 53% of potential fire resilience are located on streams designated as intermittent in this region (Appendix S1: Figure S3, Appendix S1: Table S3). Studies have found that beavers can successfully build and maintain dams in intermittent streams and often convert intermittent streams to perennial streams by raising water tables and creating flooded conditions (Gibson & Olden, 2014). While often a positive outcome that could help counteract climate‐driven aridification and novel intermittency (Carlson et al., 2024), this could be undesirable in some California streams, where intermittency creates distinct ecological communities with adaptations for surface water drying (Bêche et al., 2006; Bogan et al., 2017; Fournier et al., 2023). Finally, it is important to note that the current beaver dam capacity model results we present here represent current maximum capacity throughout this region. The next section of our study focuses on identifying where beaver restoration could maximize benefits for water storage and fire resilience on the landscape.

### Potential water storage and fire resilience benefits from beavers

We identified 10 high‐risk watersheds with both a water deficit and more than 20% of watershed areas classified as high fire risk (Figure 4). Watersheds with a high water deficit (i.e., water demand that is greater than renewable water supply) generally have a high human development footprint, such as the Upper Carson (HUC 16050201), which includes Carson City, NV, and the highly populated Lake Tahoe (HUC 16050101). On the other hand, areas with high fire risk, as defined in this study, are associated with densities of natural fuels (e.g., forests, scrub, or other vegetation), and do not take into account human development or structures at risk of fire damage (US Forest Service, 2020). Our analysis also identified areas where potential water storage and fire resilience benefits overlap (Figure 5), which would allow us to maximize the broader global change benefits of restoring beaver populations in this region. Five of the watersheds with a water deficit and high fire risk also had considerable potential water–fire benefits, suggesting that restoring beaver populations in these watersheds could create benefits in areas most at risk for the adverse effects of global change.

We note that areas with high potential water storage are not necessarily areas with the highest potential dam capacity. In this region, dam capacity tended to be highest in small headwater streams, but potential water storage was mostly concentrated in lower elevation, wide, low‐gradient streams with lower dam capacities. The geometry of these stream reaches creates more storage potential even with fewer beaver dams. In this study, we estimated that individual beaver ponds could store up to 10,000 m^3^ of surface water, with an average of 328 m^3^ (SD: 1047 m^3^). As the large SD indicates, potential water storage is highly variable and depends on geomorphic, biological, and behavioral factors not included in our models. These water storage estimates align with observations of water storage in beaver ponds around the world. Bathymetric surveys of beaver ponds have estimated that ponds store up to 1.1e10 m^3^ of surface water in total globally, with up to 9000 m^3^ of water in individual ponds and large beaver dam complexes in Washington have been shown to store almost 1000 m^3^ of water (Dittbrenner et al., 2022; Karran et al., 2017). These literature values and the water storage estimates in our study only include surface water storage, but beaver complexes also increase groundwater storage. A study in Washington state found that beaver complexes stored 2.4 times more groundwater than surface water (Dittbrenner et al., 2022), and the extent of groundwater response to beavers depends on valley width, channel confinement, and underlying soil types (Dittbrenner et al., 2022; Hill & Duval, 2009; Majerova et al., 2015; Westbrook et al., 2006). Overall, our potential water storage estimates align with other observations of surface water storage in beaver ponds, but do not include groundwater storage, which represents even greater potential for slowing and storing water.

The connection between beaver dam‐building activity and fire resilience is related to beavers creating wetlands, raising water tables, and digging water‐filled channels that fan into floodplain areas. Studies throughout the western United States have found that streams with beaver activity maintain significantly higher vegetation greenness and decreased burn severity during fire events (Fairfax et al., 2024; Fairfax & Whittle, 2020), and beavers are increasingly recognized for their potential to create fire‐resilient landscapes (Fairfax & Whittle, 2020; Jordan & Fairfax, 2022). While we restricted our fire resilience estimates to stream reaches with dam capacities of >5 dams/km, studies of megafires in the Rocky Mountains have found that the presence of any number of beaver dams reduces burn severity, regardless of BRAT‐modeled dam capacity for that stream segment (Fairfax et al., 2024). Additionally, the areas we identified as high fire risk areas are not the only areas that are susceptible to wildfires, because we only considered the highest risk areas. As such, beavers have the potential to create fire resilience anywhere on the landscape, and our estimates of where and how much beaver restoration could create fire resilience are extremely conservative.

We note that most of the high‐risk watersheds (7 out of 10) occur on the western slope of the Sierra Nevada range. We suspect this occurs because the Western Sierra is relatively more populated and receives more rainfall. Because fire risk estimates are partially based on vegetation, higher biomass build‐up in the wetter western Sierra likely explains its higher fire risk levels.

### Other considerations for prioritizing beaver reintroduction and conservation

Beavers have ecosystem‐level impacts beyond their potential for water storage and fire resilience. In particular, beaver dam‐building activity tends to benefit wetland taxa such as amphibians, waterbirds, and lentic invertebrates (e.g., water beetles and dragonflies), since they create slow‐moving habitats, wet meadows, and improve stream‐floodplain connection (e.g., Larsen et al., 2021). A variety of wetland species exist in California that could be specifically benefited by beaver ecosystem engineering. For example, the federally endangered Sierra Nevada Yellow‐Legged Frog (*Rana sierrae*) has been observed in and around beaver ponds, which create slow‐flowing lentic waters critical to this species (Brown et al., 2019; CDFW, 2024; Yarnell et al., 2019). Similarly, beaver activity has the potential to benefit the federally threatened California Tiger Salamander (*Ambystoma californiense*). There is extremely limited work on the relationship between beaver activity and *A. californiense*, but studies on Barred Tiger Salamanders (*Ambystoma mavortium*) in the Rocky Mountain region found positive relationships between beaver ponds and tiger salamanders (CDFW, 2024; Hossack et al., 2015). Overall, very little research exists about interactions between California endemic wetland species and beavers, and future work is needed to examine how beaver restoration influences biodiversity and abundance of critical wetland species. Apart from wetland species, beavers create wet meadow ecosystems and influence food availability for herbivores such as deer and elk, and may also interact with wolves and other large predators, which prey upon both deer and beavers (Gable et al., 2020). At the ecosystem level, increased aquatic primary and secondary productivity caused by slower moving water (Palmer & Ruhi, 2019) can strengthen connections between rivers and the adjacent terrestrial environments, for example via increased insect emergence and increased reliance of riparian predators (e.g., insectivorous birds, lizards, and bats) on river‐derived prey (Leathers et al., 2024). These observations underscore the need to consider how reintroducing and conserving beavers can have far‐reaching effects on ecosystems, realized via both trophic and non‐trophic mechanisms.

Restoring beavers to mountain streams is a well‐recognized strategy for managing sediment transport, increasing carbon storage, and adding large woody debris into the river system, especially in fire‐prone landscapes. Beaver‐engineered landscapes increase floodplain sediment deposition and bury particulate organic carbon; saturate soils and limit the decomposition of organic carbon; and store carbon in the form of coarse wood (Wohl et al., 2012; Wohl & Scott, 2017). Elevated carbon storage rates in beaver meadows can persist for up to three decades after beavers have abandoned the area, even though carbon storage in abandoned dams declines over time (Laurel & Wohl, 2019; Sutfin et al., 2021). Beaver ponds are also effective for storing sediment postfire, which helps reduce the harmful effects of pulsed postfire sediment on freshwater ecosystems and water sources for human communities (Dunn et al., 2024). Overall, beaver activity can increase river corridor resilience to disturbances (Rathburn et al., 2018; Wohl et al., 2024), and carbon and sediment storage management are important considerations for restoring beaver populations in fire‐prone areas of the Sierra Nevada region.

The Sierra Nevada region included in this study represents a wide variety of land ownership and management types, including public and private lands. Specifically, in the five watersheds identified as high‐risk/high potential for both drought and fire, we found that the majority of land is owned by private entities, and the majority of potential beaver dams and water storage also occurs on private lands. However, potential fire resilience tends to be more prevalent on public lands. This indicates considerable potential for the state of California to focus on beaver restoration on public lands, as well as the need to engage private landowners in beaver restoration. After beaver policy in California was updated in June 2023 to allow for beaver translocation within the state (CDFW, 2023), the California Department of Fish and Wildlife started accepting applications from land management entities and private landowners who wish to have beavers relocated to their property. In the future, incentives and cost‐sharing programs via Farm Bill conservation programs or California Wildlife Conservation Board resources could encourage additional beaver restoration activity on private lands and help mitigate any conflicts that might arise with increased beaver activity throughout the region.

Perhaps the largest impediment to the success of beaver reintroduction and persistence is livestock grazing, both on public and private lands (Small et al., 2016). Since their widespread introduction during the 19th century, livestock grazing has disproportionately affected riparian ecosystems. Over many decades, streams that once hosted stands of shade‐producing and soil‐stabilizing willows, cottonwoods, and other tree species no longer do. These tree species may still be present but are grazed down to the point of no longer having significant influence on ecosystem structure. If livestock are excluded, riparian ecosystems can recover (Fesenmyer et al., 2018; Kauffman et al., 2022). For beavers to build successful dams, there must be enough vegetation for beavers to utilize for dam construction, which requires addressing livestock grazing (Ripple et al., 2022; Small et al., 2016). On federal lands, the voluntary relinquishment of federal grazing permits in exchange for compensation, or alternatively adoption of more sustainable grazing methods like flash grazing or rotational grazing, can make former grazing allotments far more receptive—ecologically, hydrologically, vegetationally, and politically—to beaver reintroduction and persistence (e.g. Leshy & McUsic, 2008).

## CONCLUSION

Prioritization, or identifying areas with the greatest need or greatest potential for restoration success, has guided ecosystem conservation and restoration for decades (Myers et al., 2000). Considering the outsized effects of keystone species is a crucial element of building restoration priorities. As ecosystem engineers, beavers and their dam building create critical ecosystem and biodiversity benefits, as well as landscape‐scale resilience to climate change. We found that an estimated 51% of potential beaver dam capacity remains in California's Sierra Nevada compared to historical levels, and that beaver dams have the potential to store 120 million m^3^ of surface water and create 2200 km^2^ of fire resilience in areas defined as high fire risk within our study region. Specifically, we identified five watersheds that have both high risk for drought and fire impacts, and also have high potential to benefit from beaver restoration. This study used a new approach to estimating potential co‐benefits of restored beaver populations and suggests that wildlife reintroduction could help increase future climate resilience in the Sierra Nevada range.

## AUTHOR CONTRIBUTIONS

Jessie A. Moravek, Andrea Molod, Andy Kerr, Randi Spivak, Justin Brashares, Manuela Girotto, Augusto Getirana, and Albert Ruhí conceived ideas. Andrea Molod, Manuela Girotto, and Randi Spivak provided funding. Shane Feirer, Robert Johnson, and Jessie A. Moravek adapted and ran the BRAT model. Jessie A. Moravek analyzed data. Jessie A. Moravek, Justin Brashares, Albert Ruhí, and Emily Fairfax assisted with writing the manuscript. All authors contributed critically to drafts and gave final approval for publication.

## CONFLICT OF INTEREST STATEMENT

The authors declare no conflicts of interest.

## Supporting information


Appendix S1.

Appendix S2.

Appendix S3.

## Data Availability

Data and code (Moravek et al., 2025) are available on HydroShare at https://doi.org/10.4211/hs.74c8ad5a706a446a8f8d0cdfcf05e523. Additional data used in this publication are publicly available as described in Appendix S3.
