# Supplementary material for: Maximizing the potential benefits of beaver restoration for fire resilience and water storage
Source: Ecol Appl. 2025 Oct 13;35(7):e70102. doi: 10.1002/eap.70102 (PMC12518694; doi:10.1002/eap.70102)
Supplement: Supplementary file 1 — Appendix S1. [file EAP-35-e70102-s001.pdf]

Appendix S1

Ecological Applications

Maximizing the potential benefits of beaver restoration for fire resilience and water storage

Jessie A. Moravek, Justin Brashares, Manuela Girotto, Randi Spivak, Andy Kerr, Andrea Molod, Shane Feirer, Robert Johnson, Augusto Getirana, Emily Fairfax, Albert Ruhi

Figure S1: Wildfire Hazard potential across the study region.

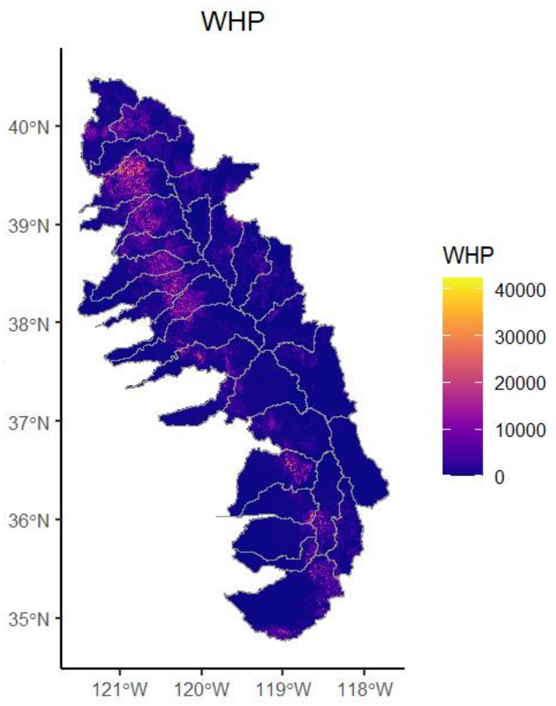

Figure S2: Change in beaver vegetation suitability index between historical and current vegetation layers. Most dam capacity loss can be attributed to major shifts in vegetation type, especially in the Central Valley area where natural vegetation has largely been converted to agricultural landscapes. Beaver-favorable vegetation has changed over time. Blue represents an increase in beaver-favorable vegetation, and red indicates a decrease.

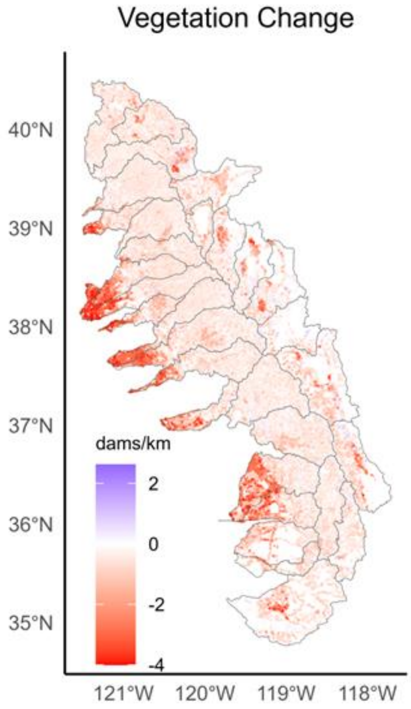

Figure S3: Potential beaver dam building capacity in intermittent streams. Intermittent streams make up 46.8% of all streams in this region. Intermittent streams store 23% of total potential water storage and create 52% of potential fire resilience area in this region, meaning that intermittent streams are disproportionately more important to fire resilience than perennial streams.

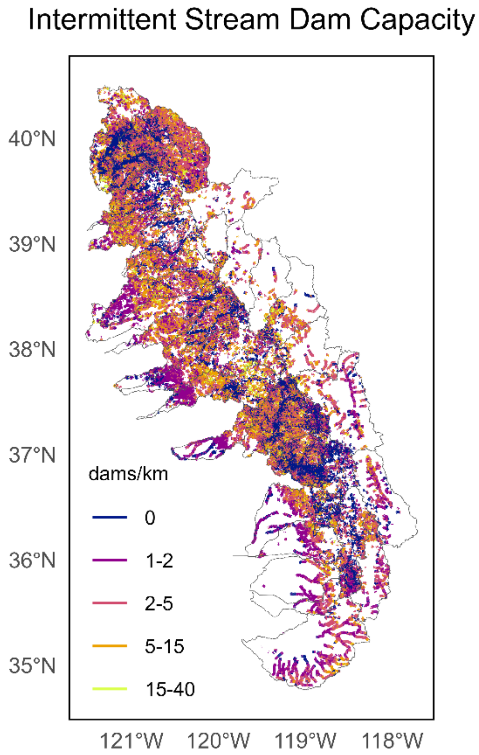

Table S1: Summary totals of current and historical dam capacity across the region.

	Number of dams	Stream length (km)
Current	897,000	159,000
Historical	440,000	159,000

Table S2: Historical and current dam capacity (dams/km), percent dam capacity remaining (%), water deficit (NDC), fire risk (% of total area where WHP > 2000), and potential water storage (m^3/km) broken down by watersheds. Watersheds identified as high risk/high potential are highlighted in gray.

HUC ID	Watershed Name	Mean Historical Dam Capacity (dams/ km)	Mean Current Dam Capacity (dams/ km)	Mean % Dam Capacity Remaining	Water Deficit (NDC)	Fire Risk (% of total area where WHP> 2000)	Potential Water Storage (m^3/km)	Potential Fire Resilience (% of stream area with fire resilience)
16050101	Lake Tahoe	14.25	6.80	52.79	32.13	1.83	1398.38	0.50
16050102	Truckee	10.30	5.11	55.51	1.77	20.67	3760.23	2.52
16050201	Upper Carson	9.70	4.50	58.38	159.14	21.57	3755.90	2.09
16050301	East Walker	10.95	5.75	68.29	3.33	12.91	3393.05	1.11
16050302	West Walker	10.30	4.83	64.60	2.80	13.94	3461.71	0.65
18020121	North Fork Feather	10.48	5.00	52.50	0.64	19.50	1105.71	7.57
18020122	East Branch North Fork Feather	10.75	4.56	55.51	0.00	49.75	1432.34	23.61
18020123	Middle Fork Feather	9.80	4.41	60.16	0.44	30.05	1301.39	13.11
18020125	Upper Yuba	8.84	4.37	62.84	2.05	56.63	733.39	27.66
18020126	Upper Bear	13.09	5.21	51.80	2.60	29.54	2173.92	11.97
18020128	North Fork American	10.08	4.25	52.04	0.08	50.28	1013.67	19.56
18020129	South Fork American	12.60	5.50	50.78	1.35	41.66	854.36	15.49

18030001	Upper Kern	6.99	3.43	70.06	2.06	20.44	627.65	8.95
18030002	South Fork Kern	8.59	5.02	81.31	2.41	19.59	2389.69	2.61
18030003	Middle Kern	6.04	1.83	55.90	9.03	19.92	2429.41	1.48
18030004	Upper Poso	8.33	2.94	46.04	1.98	11.97	2721.92	2.29
18030005	Upper Deer-Upper White	5.95	1.77	44.29	3.41	3.77	3593.69	0.55
18030006	Upper Tule	9.49	2.49	30.52	1.78	7.77	2336.68	2.04
18030007	Upper Kaweah	9.33	2.82	37.33	13.40	17.11	1871.34	5.68
18030010	Upper King	6.40	4.09	80.44	2.45	13.57	491.87	8.67
18040006	Upper San Joaquin	7.30	4.74	81.00	3.89	8.84	506.75	4.39
18040007	Fresno River	9.06	3.11	38.99	2.21	9.06	2528.12	3.72
18040008	Upper Merced	11.54	5.37	54.74	5.94	20.44	1116.04	5.79
18040009	Upper Tuolumne	10.79	4.50	61.29	8.25	12.23	1526.60	3.80
18040010	Upper Stanislaus	10.72	3.89	44.24	15.49	29.29	888.22	11.41
18040011	Upper Calaveras California	15.68	4.46	33.45	4.83	19.13	2317.61	5.39
18040012	Upper Mokelumne	15.28	1.98	19.64	25.27	26.23	1459.40	8.91
18040013	Upper Cosumnes	13.69	4.30	38.45	6.07	35.75	3193.78	13.83

18090101	Mono Lake	10.86	6.85	104.51	6.06	6.66	1679.80	0.38
18090102	Crowley Lake	9.60	3.79	80.98	6.19	5.70	3300.41	0.24
18090103	Owens Lake	10.53	3.34	78.84	0.00	0.23	2466.65	0.07

Table S3: Current potential dam capacity broken down by percent of stream that is intermittent. The rest of the stream is perennial.

Dam Capacity	% intermittent (by length)
All streams	47.5%
0 dams/km	12.8%
1-2 dams/km	0.7%
2-5 dams/km	16.7%
5-15 dams/km	9.2%
15-40 dams/km	1.5%

Table S4: Private land and potential beaver dams, water storage, and fire resilience on private lands in each watershed. Watersheds identified as high risk/high potential are highlighted in gray.

HUC ID	Watershed Name	% Private Land	% Potential Dams on Private Land	% Potential Water Storage on Private Land	% Potential Fire Resilience on Private Land
16050101	Lake Tahoe	34.7	17.5	55.9	1.1
16050102	Truckee	14.1	30.7	19.4	9.2
16050201	Upper Carson	4.2	15.1	8.5	11.3
16050301	East Walker	6.7	21.8	8.4	7.2
16050302	West Walker	4.1	18.6	22.2	0.4
18020121	North Fork Feather	44.3	49.8	63.9	16.7
18020122	East Branch North Fork Feather	19.4	32.9	56.0	8.1
18020123	Middle Fork Feather	37.7	46.4	78.9	13.1
18020125	Upper Yuba	47.9	66.4	72.7	18.5
18020126	Upper Bear	89.1	91.5	92.7	44.6
18020128	North Fork American	34.5	45.9	40.3	17.2
18020129	South Fork American	49.1	57.3	61.3	34.7
18030001	Upper Kern	2.3	6.2	9.3	2.2
18030002	South Fork Kern	9.5	10.5	26.5	1.6

18030003	Middle Kern	80.7	82.3	97.2	14.1
18030004	Upper Poso	89.5	81.6	98.2	27.4
18030005	Upper Deer-Upper White	92.6	78.3	81.7	23.8
18030006	Upper Tule	71.6	63.8	93.2	10.2
18030007	Upper Kaweah	70.7	58.9	90.0	13.5
18030010	Upper King	5.8	10.1	12.6	4.8
18040006	Upper San Joaquin	13.7	13.0	22.0	18.4
18040007	Fresno River	92.2	72.7	95.0	34.4
18040008	Upper Merced	29.8	24.3	43.2	10.6
18040009	Upper Tuolumne	35.4	33.1	54.3	21.1
18040010	Upper Stanislaus	40.3	37.2	54.2	18.4
18040011	Upper Calaveras California	89.4	92.0	97.6	49.6
18040012	Upper Mokelumne	71.9	70.8	87.7	30.3
18040013	Upper Cosumnes	78.9	77.5	88.8	33.1
18090101	Mono Lake	4.0	19.3	42.4	15.8
18090102	Crowley Lake	3.2	10.3	12.3	3.0
18090103	Owens Lake	11.0	6.6	5.1	0.0
