# Supplementary material for: Maximizing the potential benefits of beaver restoration for fire resilience and water storage
Source: Ecol Appl. 2025 Oct 13;35(7):e70102. doi: 10.1002/eap.70102 (PMC12518694; doi:10.1002/eap.70102)

Appendix S2

Ecological Applications

Maximizing the potential benefits of beaver restoration for fire resilience and water storage

Jessie A. Moravek, Justin Brashares, Manuela Girotto, Randi Spivak, Andy Kerr, Andrea Molod, Shane Feirer, Robert Johnson, Augusto Getirana, Emily Fairfax, Albert Ruhi

Table S1: Preliminary BRAT validation in four Sierra Nevada watersheds for stream reaches where beaver dams are present. These four watersheds were chosen because they represent different California ecoregions, and they have active beaver populations. Beaver dams were mapped using high resolution satellite imagery throughout the entirety of all 4 watersheds, and only reaches with beaver dams present were assessed. We only assessed reaches with beaver dams because BRAT is a maximum capacity model, meaning the only way to validate the model is to compare model results with places that have active beaver dams. The BRAT model bins potential dam capacity (a continuous variable) into five categories (None—0 dams/km; Rare—0-1 dams/km; Occasional—1-5 dams/km; Frequent—5-15 dams/km; and Pervasive—15+ dams/km). We assessed whether actual beaver dam counts in each reach matched the predicted BRAT category. In most watersheds, BRAT underestimates or correctly estimates the dam capacity of most stream reaches. In some watersheds BRAT overestimates dam capacity, perhaps because beavers in those reaches do not persist at capacity.

Watershed	# of reaches with BRAT overestimate	# of reaches with BRAT underestimate	# of reaches with accurate estimate
HUC 18030003	0	4	0
HUC 18030002	12	67	40
HUC 18090101	3	6	2
HUC 18020123	10	20	11

Figure S1: An example of validating BRAT model results in the Sierra Nevada region. This map shows hand-mapped beaver dams (blue lines, panel A) in the Lundy canyon of the Mono Lake watershed (HUC 18090101, panel B) compared to BRAT model estimates (colorful lines). We assessed model under- or over-estimates if the number of dams within a 100m buffer of each stream reach did not align with the binning category predicted by the model. In this example, BRAT model underestimates beaver dam capacity (in this case, reaches are classified as frequent when hand-mapped dams indicate they should be marked as pervasive)

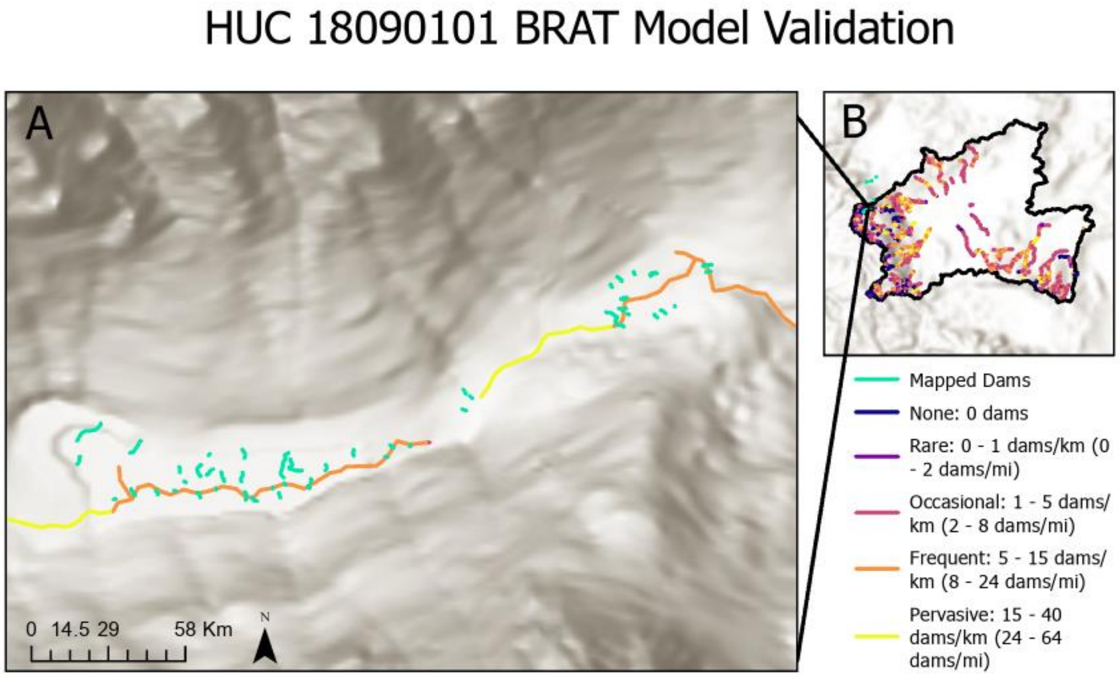

Supplement: Supplementary file 2 — Appendix S2. [file EAP-35-e70102-s003.pdf]
