# Supplementary material for: Maximizing the potential benefits of beaver restoration for fire resilience and water storage
Source: Ecol Appl. 2025 Oct 13;35(7):e70102. doi: 10.1002/eap.70102 (PMC12518694; doi:10.1002/eap.70102)
Supplement: Supplementary file 3 — Appendix S3. [file EAP-35-e70102-s002.pdf]

Appendix S3

Ecological Applications

Maximizing the potential benefits of beaver restoration for fire resilience and water storage

Jessie A. Moravek, Justin Brashares, Manuela Girotto, Randi Spivak, Andy Kerr, Andrea Molod,

Shane Feirer, Robert Johnson, Augusto Getirana, Emily Fairfax, Albert Ruhi

Additional data used in this publication are publicly available as described herein.

“BLM National SMA Surface Management Agency Area Polygons.” 2024. US Department of the Interior Bureau of Land Management. April 18, 2024. https://gbp-blm-egis.hub.arcgis.com/datasets/6bf2e737c59d4111be92420ee5ab0b46/about.

Devineni, Naresh, Upmanu Lall, Elius Etienne, Daniel Shi, and Chen Xi. 2015. “America’s Water Risk: Current Demand and Climate Variability.” Geophysical Research Letters 42 (7): 2285–93. https://doi.org/10.1002/2015GL063487.

Landfire. 2021. “LANDFIRE Map Viewer.” Department of Interior, U.S. Geological Survey, and U.S. Department of Agriculture. https://www.landfire.gov/viewer/.

Landfire Existing Vegetation Type and Biophysical Settings datasets were downloaded from www.landfire.gov/viewer/. To access these data:

1. At the top of the map window, click the “select download” button.
    a. This will open the “Data Download Tool” menu on the right side of the screen. Under “Method”, open the dropdown menu and select “Template”
2. Open the dropdown menu and select “Atlas State Boundaries” .
3. Select state of interest.
4. In the drop-down menu on the right side of the screen, expand “LF 2022 (LF_230)”, then “Vegetation”, then check “us_230 Existing Vegetation Type”. Then expand “LF 2020 (LF_220)”, then “Vegetation”, then check “us_220 Biophysical Settings”.
5. Enter email. Click “Download”.

Riverscapes Consortium. 2018. “BRAT.” California BRAT iHyd Equations. 2018. https://brat.riverscapes.net/Documentation/Standards/Specific%20Projects/TNC_BRAT_Notes.html.

Low and high flow regression equations were obtained from https://brat.riverscapes.net/Documentation/Standards/Specific%20Projects/TNC_BRAT_Notes.html. To access these data, click the link under "iHyd" for a spreadsheet of basin-specific regression equations.

United States Census Bureau. 2023. "TIGER/Line® Shapefiles." Shapefile dataset. https://www.census.gov/cgi-bin/geo/shapefiles/index.php.

Road and railroad shapefiles were obtained from https://www.census.gov/cgi-bin/geo/shapefiles/index.php. To access these data, select "roads" and "rails" under "select data type". Railroads can be downloaded in a national file. Primary and secondary roads can be downloaded by county.

U.S. Forest Service. 2020. "Wildfire Hazard Potential | Missoula Fire Sciences Laboratory." USDA Forest Service, Fire Modeling Institute. https://research.fs.usda.gov/firelab/products/dataandtools/wildfire-hazard-potential.

U.S. Geological Survey. 2019. "The StreamStats Program." 2019. https://streamstats.usgs.gov/ss/.

Basin-specific inputs were obtained from https://streamstats.usgs.gov/ss/. To access these data:

1. Select state of interest
2. Identify the most downstream part of the watershed of interest and zoom in to level 15 or greater.
3. Click the "Delineate" button on the left side of the screen, then place your cursor on the most downstream pixel in your watershed.
4. If needed, use the "Edit Basin" tool on the left.
5. Click "Continue". Under "Regression Based Scenarios" select all three. This will automatically select "Basin Characteristics".
6. Scroll down and click "Continue" then click "Open Report". This will open a pop-up window with the necessary basin-specific inputs.

U.S. Geological Survey. 2024. "3D Elevation Program." 2024. https://www.usgs.gov/3d-elevation-program.

Digital Elevation Models were obtained from https://www.usgs.gov/3d-elevation-program. To access these data:

1. Access https://apps.nationalmap.gov/downloader/
2. Expand "Elevation Products (3DEP)"
    a. Under Subcategories, select ⅓ arc-second DEM, Current
    b. Under File Formats, select GeoTiff

3. At the top, next to “Area of Interest”, expand the drop down menu on “Map Extent/Geometry”. Select “Selectable Polygon”
4. Once you click “Selectable Polygon”, you will see a “Select” with a dropdown menu. Choose “8-digit HU (Subbasin)” from the dropdown menu.
5. Click your watershed of interest to highlight it and click “Search Products”
6. You will see DEM tiles that overlap your watershed of interest.
7. Click the shopping cart icon next to each needed tile.
8. Click on “Cart” in the top menu
9. Download the selected .tiff files

U.S. Geological Survey. 2024. “National Hydrography Dataset.” 2024. https://www.usgs.gov/national-hydrography/national-hydrography-dataset.

U.S. Geological Survey. 2024. “Protected Areas Database of the United States (PAD-US) 4.0.” U.S. Geological Survey (USGS) Gap Analysis Project (GAP). https://doi.org/10.5066/P96WBCHS.
